# Measurement System for Current Transformer Calibration from 50 Hz to 150 kHz Using a Wideband Power Analyzer

**DOI:** 10.3390/s25175429

**Published:** 2025-09-02

**Authors:** Mano Rom, Helko E. van den Brom, Ernest Houtzager, Ronald van Leeuwen, Dennis van der Born, Gert Rietveld, Fabio Muñoz

**Affiliations:** 1 VSL B.V. (VSL), 2629 JA Delft, The Netherlands; 2 Electrical Sustainable Energy Department, Delft University of Technology (TU Delft), 2628 CD Delft, The Netherlands; 3 Department of Electrical Engineering, Mathematics and Computer Science (EEMCS), University of Twente, 7522 NB Enschede, The Netherlands

**Keywords:** current transformers, current ratio, wideband, wide dynamic range, calibration, precision power analyzers, sampling current ratio bridge, phase error, ratio error, uncertainty

## Abstract

Accurate and reliable characterization of current transformer (CT) performance is essential for maintaining grid stability and power quality in modern electrical networks. CT measurements are key to effective monitoring of harmonic distortions, supporting regulatory compliance and ensuring the safe operation of the grid. This paper addresses a method for the characterization of CTs across an extended frequency range from 50 Hz up to 150 kHz, driven by increasing power quality issues introduced by renewable energy installations and non-linear loads. Traditional CT calibration approaches involve measurement setups that offer ppm-level uncertainty but are complex to operate and limited in practical frequency range. To simplify and expand calibration capabilities, a calibration system employing a sampling ammeter (power analyzer) was developed, enabling the direct measurement of CT secondary currents of an unknown CT and a reference CT without any further auxiliary equipment. The resulting expanded magnitude ratio uncertainties for the wideband CT calibration system are 10 ppm (k=2) up to 10 kHz and less than 120 ppm from 10 kHz to 150 kHz; these uncertainties do not include the uncertainty of the reference CT. Additionally, the operational conditions and setup design choices, such as instrument warm-up duration, grounding methods, measurement shunt selection, and cable type, were evaluated for their impact on measurement uncertainty and repeatability. The results highlight the significance of minimizing parasitic impedances at higher frequencies and maintaining consistent testing conditions. The developed calibration setup provides a robust foundation for future standardization efforts and practical guidance to characterize CT performance in the increasingly important supraharmonic frequency range.

## 1. Introduction

High-frequency distortion in electricity grids is gaining scientific and technical interest due to its growing occurrence and impact. These disturbances are mainly injected by new devices for decentralized renewable generation. Power converters connected to the grid increase switching frequencies, introducing harmonic components into the grid [1,2]. These harmonics, produced by non-linear loads and switching devices, degrade power quality (PQ). Recent studies report reductions in the power factor (up to 60%) and increases in line losses exceeding 2% [3,4]. Although international standards such as IEC 61000-3-2 [5] and IEEE Std 519 [6] provide harmonic emission limits, enforcement remains inconsistent, emphasizing the need for enhanced PQ monitoring.

Measurements in medium-voltage (MV, up to 36 kV) distribution networks typically rely on instrument transformers (ITs), such as current transformers (CTs), to scale down currents (up to 2 kA) for accurate evaluation by PQ analyzers and power analyzers. Standardization for PQ analyzers is well-established up to 150 kHz and 20 A at 230 V [7,8]. Extensive research has refined the measurement methods aligned with these standards [9,10]. In contrast, the performance of instrument transformers at higher frequencies, especially in the supraharmonic range (that is, in the range of 2–150 kHz), remains less well defined [11]. Current standards like IEC 61869-2 [12] and recent research [13,14] address performance up to lower-order harmonics but do not cover frequencies beyond 10 kHz, where particular distortions occur as well [15,16].

Supraharmonic distortions pose risks to medium-voltage distribution grids [17]. High dV/dt voltage spikes exceeding 100 kV/μs can lower partial-discharge inception voltages, potentially shortening insulation lifetimes by as much as an order of magnitude [17,18,19]. Moreover, supraharmonics increase eddy-current and proximity losses in transformers, which can raise hotspot temperatures by up to 10–15 °C [20]. These distortions can trigger unintended activation of protective devices, posing a risk to grid stability, and generate audible noise exceeding 50 dB(A) in low-voltage switchgears [19]. Additionally, supraharmonic distortion can propagate from medium-voltage networks into low-voltage circuits through transformers, causing LED lighting flicker, interference with power-line communication, and damage to domestic appliances and sensitive equipment [17,21].

To address the risks associated with increasing harmonic emissions in medium-voltage grids, it is essential to regulate the generation of these emissions and ensure that supraharmonic voltages and currents can be accurately measured. Consequently, wideband accuracy classes (WB0–WB4) for CTs have been proposed in IEC 61869-2 [12] for frequencies up to 500 kHz, but the metrological infrastructure needed to verify such performance is still incomplete. In response, National Metrology Institutes (NMIs) have extended CT calibration services into the wideband region. Several leading laboratories now offer CT characterizations up to 10 kHz. For example, the comparator system described in [22] achieved expanded uncertainties (k=2) of a few hundred parts per million (ppm) for magnitude ratio error and a few microradians for phase measurements up to 9 kHz. Similarly, the reference setup developed in [23] demonstrated uncertainties of ±20 ppm and ±20 μrad at the fundamental frequency, rising to ±400 ppm and ±800 μrad at 9 kHz.

Despite the recent progress, several important limitations remain. Most current measurement systems have only been validated up to 10 kHz, leaving the frequency range between 10 kHz and 150 kHz largely uncharacterized and unexplored [23]. In addition, the existing wideband calibration techniques often depend on auxiliary equipment, such as wideband shunts, current transducers, and buffer amplifiers [24,25,26], which complicates the calibration process, especially at higher frequencies; see also Section 2.2.2.

The sampling ammeter approach introduced in [27] offers a promising simplification by reducing the number of required components and streamlining the procedure. However, this system was only evaluated up to 10 kHz and did not provide a complete analysis of the measurement chain or assess the impact of practical setup variations. As a result, a comprehensive uncertainty budget for the method was not established. These limitations highlight the ongoing need for research and development in wideband CT calibration, particularly in the supraharmonic frequency band.

This paper addresses these shortcomings by introducing a broadband calibration setup capable of characterizing CT ratio and phase errors from 50 Hz to 150 kHz. Building on the sampling ammeter concept of [27], the present work uses the same measurement system and demonstrates its simplification compared to traditional wideband calibration methods that rely on more auxiliary equipment. This paper also analyzes how practical design choices—including CT proximity, conductor placement, secondary-side cabling, grounding schemes, shunt selection, and compensation electronics—influence overall measurement accuracy. In doing so, this work aims to help establish a metrological foundation for the future implementation of the IEC WB3 class [12] and supports the measurement of supraharmonic power quality phenomena.

The remainder of this paper is organized as follows. Section 2 provides the theoretical background relevant to CT ratio and phase error characterization, including the complex ratio error formulation, the ratio-based calibration approach, and an overview of the existing techniques along with their limitations. Section 3 then describes the experimental setup and methodology, outlining the measurement system architecture, instrumentation, and data processing procedures. Section 4 presents and analyzes the experimental results, covering the baseline calibration performance and examining the influence of the key operational and setup parameters. This section also discusses the main sources of measurement uncertainty and provides an uncertainty budget. Finally, Section 5 discusses the findings in the context of CT calibration practices, offers practical recommendations for wideband measurements, and suggests directions for future research.

## 2. Background and Theory

### 2.1. Complex Ratio Error of a Current Transformer

Current transformers are used for scaling high primary currents in the MV grid to lower more easily measurable secondary currents. Ideally, a CT provides a reproduction $I_{s}$ of the primary current $I_{p}$ at its secondary winding with a constant ratio $n=I_{p}/I_{s}$. However, in reality, CTs have amplitude and phase errors that can be expressed as a complex ratio error:

(1) $$\epsilon \left(\omega \right)=\frac{n\;I_{s}\left(\omega \right)}{I_{p}\left(\omega \right)}-1.$$

This ratio error can be measured as a function of frequency ω=2πf.

### 2.2. Calibration Methods for Current Transformers

Historically, instrument transformer calibration has been performed at power frequencies of 50 Hz or 60 Hz using bridge or comparator techniques [28,29,30,31]. In these configurations, a test CT and a multi-winding reference are energized by a common primary conductor, and their secondaries are connected in opposition; the residual current provides a direct measure of CT error [28]. By nulling this residual, the system determines both ratio and phase error. Although such approaches can deliver sub-ppm uncertainties, they are complex and typically limited to comparing CTs with equal nominal ratios.

The proliferation of harmonics, interharmonics, and supraharmonics in modern grids has driven the need for CT calibration over an extended frequency range [25]. Multiple wideband calibration strategies have emerged in response.

#### 2.2.1. Shunt-Based Methods

As demonstrated for instance by [25], CT calibration can be achieved using wideband shunts [32,33,34] to directly measure the primary current that is wound multiple times through the CT core. A composite signal—containing the fundamental and harmonics up to 5 kHz—is injected into the primary windings. Two wideband coaxial shunts measure primary and secondary currents simultaneously, with frequency-domain analysis providing ratio and phase error for each spectral component.

However, this approach faces practical constraints at high current and frequency. In [26], the use of coaxial manganin shunts demonstrates DC uncertainty of 10 ppm up to 10 kA but is limited to tens of kHz. In [35], experimental results are limited to below 100 A, with tens of ppm error at 100 kHz. Similarly, Ref. [36] reports uncertainties of 200 ppm to 500 ppm in amplitude and below 0.05mrad in phase up to 100 kHz at 100 A. As a result, no available shunt technology combines >1 kA rating, bandwidth to 150 kHz, and ppm-level accuracy. While research continues, the reference CT and DUT CT combination for high-current wideband calibration remains a practical alternative.

#### 2.2.2. Sampling Current Techniques

**Figure 1:**
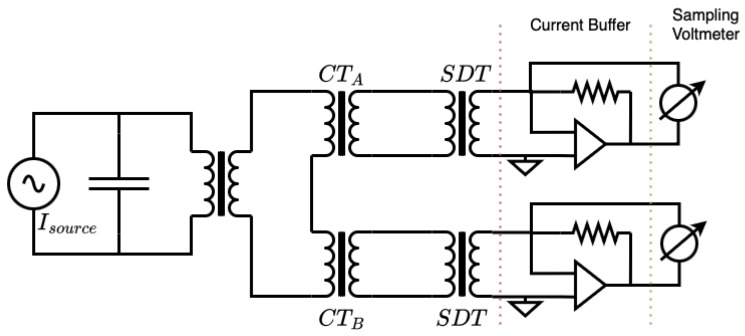
Schematic of a CT sampling current ratio measurement system. High primary currents are generated using a power amplifier and a step-up transformer. The CT under test (DUT) is measured against a reference CT where both secondary currents are scaled down using step-down transformers (SDTs). Operational amplifiers with precision AC resistors act as current buffers that convert current to voltage, while digital sampling voltmeters record the buffer voltage signals [24].

As described in [24,37], another approach employs current transducers, current buffers, and high-precision sampling voltmeters (see Figure 1 for a schematic overview of the setup used in [24]). Although such setups can achieve uncertainties at the ppm level, they are complex to maintain and operate due to the number of components, and the measurement bandwidth is ultimately limited by the particular components.

An alternative simplified measurement system introduced by [27] uses a high-resolution sampling ammeter (power analyzer) to measure secondary currents of both DUT and reference CT. It achieved uncertainties below 35 ppm up to the 50th harmonic (2 kHz), with demonstrated performance to 10 kHz, all with significantly reduced system complexity compared to earlier methods.

In this study, the digital sampling comparator measurement system from [27] is extended to 150 kHz, a comprehensive uncertainty budget is presented, and the influence of practical test-bench parameters—including grounding, cabling, conductor placement, and compensation electronics—is systematically analyzed.

Both the DUT and the reference CTs are of the type described in [38], which employ electronic compensation to achieve uncertainties below 1 ppm at 50 Hz. Minor differences in the components of the compensation electronics have shown to provide negligible impact at 50 Hz but might introduce different frequency responses. While their high-frequency performance is discussed, the development of a detailed uncertainty budget for the CTs themselves—and potential improvements to their design—are considered topics for future work. The primary focus of this paper is on the measurement system and methodology surrounding these CTs rather than on the CTs’ intrinsic properties.

### 2.3. Ratio-Based Approach for CT Characterization

Instead of measuring $I_{p}$ with a wideband high-current shunt, the device-under-test CT (DUT, subscript *X*) is placed in series with a well-characterized reference CT (subscript *R*). Because both CTs measure the same primary current, it is possible to eliminate $I_{p}$ from the equations:

(2) $$I_{sX}=\frac{I_{p}}{n_{X}}\left[1+\epsilon_{X}\left(\omega \right)\right],\;I_{sR}=\frac{I_{p}}{n_{R}}\left[1+\epsilon_{R}\left(\omega \right)\right];$$

their complex ratio provides

(3) $$\epsilon_{X}\left(\omega \right)=\frac{n_{X}}{n_{R}}\frac{I_{sX}}{I_{sR}}\left[1+\epsilon_{R}\left(\omega \right)\right]-1.$$

Hence, the error $\epsilon_{X}$ of the unknown CT can be expressed directly in terms of the measured ratio of the secondary currents and the known error $\epsilon_{R}$ of the reference CT.

## 3. Measurement Setup and Methodology

To accurately characterize current transformers over a frequency range from 50 Hz to 150 kHz, two complementary measurement approaches were employed. The primary approach involves comparing the secondary currents of the CT under test (device under test, DUT) directly against those of a reference CT. By comparing these two measurements, the ratio between the DUT and reference CT can be determined using Equation (3). Changes made to the measurement setup—such as different cable configurations—will be reflected in variations of this ratio, providing insights into the specific influence of each change.

The second approach involves directly measuring the primary current using an ammeter and comparing it to the CT’s secondary current. Although this direct measurement provides an immediate indication of the absolute ratio error of the CT, it carries greater uncertainty due to calibration of the ammeter’s gain. Nevertheless, this method remains valuable as it quantifies the true performance and absolute error of the CT. Due to difficulties in calibrating high-current high-bandwidth shunt, this method is practically limited to around 20 A.

**Figure 2:**
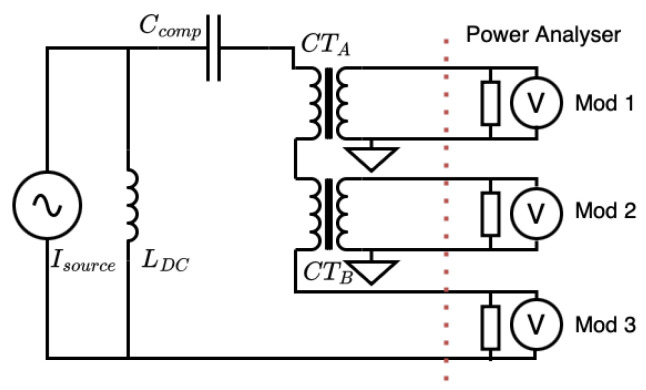
Schematic of standard CT characterization setup. A high-frequency high-current source simultaneously energizes the DUT and reference CTs, enabling relative secondary current measurement and ratio comparison.

Figure 2 illustrates the simultaneous characterization approach, where a high-frequency high-current amplifier generates up to 10 A across the specified frequency range through series-connected DUT and reference CTs. Secondary currents are measured by internal shunts within the sampling ammeter.

### 3.1. Measurement Components and Conditions

The measurements were carried out within a temperature-controlled laboratory (23.0 °C ± 0.5 °C, 45 ± 5% RH), enclosed in a Faraday cage where instruments use a 58 Hz power supply to minimize mains interference. It should be noted that these conditions do not fully mimic real-world applications where multiple influence factors such as temperature, mechanical vibration, burden, adjacent phases, and proximity effects can influence the accuracy of the CT under test [39,40]. The setup and experiments described in this paper are designed for the calibration of CTs that are used as reference CTs for further applications.

The measurement system comprises two nearly identical NRC electronically compensated current transformers [38], each with an accuracy of around 1 ppm at 50 Hz. One transformer serves as the DUT, while the other acts as the reference. These CTs use internal compensation electronics that minimize magnetization currents, improving linearity. Although the compensation electronics ensure low errors—less than 20 ppm and 20 μrad—up to approximately 5 kHz, the performance at higher frequencies is less known. In particular, the bandwidth of the internal amplifiers limits the effectiveness of compensation beyond several kilohertz. Subtle differences in amplifier design between the two models could also influence high-frequency performance. Their performance is examined throughout this paper, particularly in Section 4.8. As such, a question addressed in this study is up to what frequency the electronic compensation remains effective.

A bus-bar cable carries the primary excitation current, which is routed sequentially through the cores of both CTs to ensure exposure to identical current waveforms. The primary conductor is carefully centered in each CT using non-conductive magnetically neutral plastic spacers, standardizing the geometry and minimizing any possible position-dependent measurement effects. Although subsequent tests revealed minimal sensitivity to conductor position (see Section 4.9), this arrangement allows for the separation of variables.

Current and voltage measurements are performed using a precision power analyzer (WT5000 from Yokogawa Electric Corporation, Tokyo, Japan), capable of high-speed sampling up to 2 MS/s. The analyzer contains interchangeable modules equipped with precision shunt resistors: 6.5 mΩ (up to 30 A), 110 mΩ (up to 5 A), and 500 mΩ (up to 500 mA). The appropriate shunt selection and its impact on measurement uncertainty are detailed in Section 4.6. Throughout this work, the term “sampling ammeter” refers to this instrument.

The sampling ammeter integrated within the power analyzer was calibrated to ensure accurate current measurements. First, calibration of the voltage channels was performed using an AC measurement standard (5790B from Fluke Corporation, Everett, WA, USA), which provided a reference voltage to the power analyzer voltage input. For current calibration, the standard measurement circuit was used to generate either 100 mA or 10 A through precision shunt resistors (type JV; see [34]). Both the voltage and current channels were recorded simultaneously, and the amplitudes were determined using the same signal processing algorithm as applied in the main experimental measurements, minimizing systematic bias.

This calibration procedure results in an estimated gain uncertainty of approximately 2.5 ppm at 50 Hz and up to 50 ppm at 150 kHz for the power analyzer. It should be noted that, in the secondary-to-secondary current comparison, this uncertainty is effectively eliminated due to the interchange of the measurement channels between runs.

Measurement data is collected, stored, and processed on a laptop PC, which interfaces with the sampling ammeter via high-speed USB for transfer of raw measurement data.

Twisted-pair cables are used to connect the secondary outputs of both CTs to the sampling ammeter, minimizing induced electromagnetic interference, as discussed in Section 4.11. For diagnostic and performance verification, additional cables are connected to directly measure the secondary voltage at the CT terminals.

All secondary circuits, the ammeter enclosure, the CT compensation electronics, CT housings, and the high-current generation system are connected to a single common ground point on the ammeter. This single-point grounding strategy is implemented to prevent ground loops and minimize electrical noise, as described in Section 4.5.

### 3.2. High-Bandwidth Power Amplifier System

To generate the required primary excitation current of 10 A across the full frequency range up to 150 kHz, a dedicated high-bandwidth amplifier system was employed. The core of the setup is an arbitrary waveform generator, which provides a stable sinusoidal output with controllable frequency. This signal is passed into an adjustable potentiometer, enabling manual smooth ramp-up of the current amplitude. This allows for consistent primary current levels at each frequency while also providing an additional layer of safety. This signal is then fed to a high-bandwidth amplifier capable of delivering the necessary power to drive high currents through the CTs, overcoming the frequency-dependent reactance within the measurement circuit.

To ensure that no direct current component is present, which could otherwise lead to unwanted magnetization of the CT cores, auxiliary LC filtering is implemented. An inductor serves as a DC bypass, while a series-connected capacitor compensates for circuit reactance at higher frequencies.

For quick verification of the generated primary current, a clamp meter is placed around the primary current carrying bus-bar through the CTs, which is used to confirm the amplitude prior to and during each test.

### 3.3. Gain Elimination Method for Sampling Ammeter

When the same primary current $I_{p1}$ is fed through both CTs, each CT’s secondary current is measured on a separate current ammeter channel (or “module”). Let $g_{\mathrm{mod}1}$ and $g_{\mathrm{mod}2}$ be the gain correction factors of these two modules (ideally $g_{\mathrm{mod}}=1$). The measured secondary currents are then

(4) $$I_{CTA1}=I_{p1}\;\;\cdot \;\;n_{CTA}\;\;\cdot \;\;g_{\mathrm{mod}1},\;I_{CTB1}=I_{p1}\;\;\cdot \;\;n_{CTB}\;\;\cdot \;\;g_{\mathrm{mod}2}\;.$$

Because $g_{\mathrm{mod}1}$ and $g_{\mathrm{mod}2}$ are never truly unity and vary with factors like frequency and ammeter configuration, one cannot directly infer $n_{CTA/B}$ from either $n_{CTA/B1}$ or $n_{CTA/B2}$ alone. At the ppm level, measuring and compensating for these gains across the entire frequency range would be cumbersome.

A method to eliminate module gain errors is to interchange the measurement connections: after the initial measurement, CT A is connected to the second current ammeter module, and CT B is connected to the first module. A second primary current $I_{p2}$ (which will be approximately the same as $I_{p1}$) is then applied, yielding the ratio measurements

(5) $$\begin{array}{cc} n_{CTA/B\;1} & =\frac{I_{CTA\;1}}{I_{CTB\;1}}=\frac{n_{CTA}\;g_{\mathrm{mod}1}}{n_{CTB}\;g_{\mathrm{mod}2}}\;\;\;\mathrm{and}\;\;\;n_{CTA/B\;2}=\frac{I_{CTA\;2}}{I_{CTB\;2}}=\frac{n_{CTA}\;g_{\mathrm{mod}2}}{n_{CTB}\;g_{\mathrm{mod}1}}, \end{array}$$

where the second equation applies after interchanging the modules.

The unknown module gains appear in reciprocal form across the two ratio measurements, $n_{CTA/B\;1}$ and $n_{CTA/B\;2}$. By taking the geometric mean, the module gains cancel out:

(6) $$n_{CTA/B}=\sqrt{\;n_{CTA/B\;1}\;\;\cdot \;\;n_{CTA/B\;2}}=\sqrt{\;\frac{n_{CTA}\;g_{\mathrm{mod}1}}{n_{CTB}\;g_{\mathrm{mod}2}}\times \frac{n_{CTA}\;g_{\mathrm{mod}2}}{n_{CTB}\;g_{\mathrm{mod}1}}}=\frac{n_{CTA}}{n_{CTB}}.$$

This simple interchanging approach therefore provides the relative ratio $n_{CTA/B}$ without individually characterizing the module gains. In practice, this technique simplifies the calibration of CTs.

### 3.4. Ratio Estimation Algorithm

Accurate estimation of amplitude and phase for discrete sinusoidal signals is crucial for precise CT ratio measurements. To achieve this, it is important to use good measurement practices, such as selecting a sampling frequency that is an exact integer multiple of the signal frequency, thoroughly removing any DC offset, and acquiring sufficiently long data records. By taking a Fast Fourier Transform (FFT) of the signal and selecting the highest bin, the signal amplitude is estimated from the magnitude of this dominant frequency bin. However, when the frequency of the signal does not exactly align with discrete FFT bins, spectral leakage occurs. This leakage redistributes signal energy into adjacent bins, causing inaccurate amplitude estimates. It is important to note that, technically, if one measures the ratio between two signals with small phase differences using the highest-bin FFT method, amplitude biases may partially cancel out. Moreover, this approach does not accurately estimate the absolute amplitude of the sine wave, preventing verification of the expected measurement results.

A solution is the use of a resampling algorithm [41]. This method effectively realigns signal frequencies onto FFT bins, reducing leakage and simplifying analysis. Despite its effectiveness, resampling is computationally very intensive. As an alternative, this paper uses the interpolated Discrete Fourier Transform (DFT) technique, specifically the three-point interpolated Hanning window DFT method (idft3phann), based on [42] and adapted from the algorithm in [43].

#### Algorithm Steps

**Figure 3:**
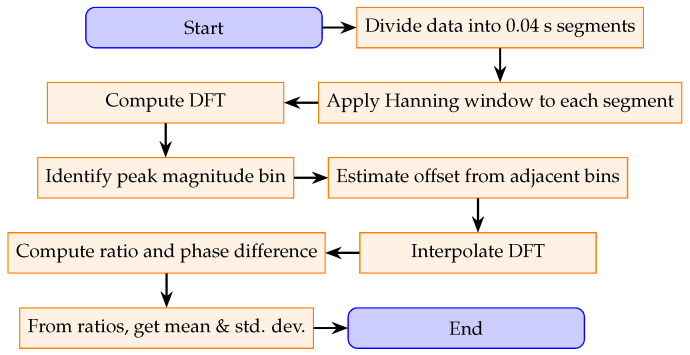
Flowchart of the algorithm for determining the ratios.

The process for determining the ratio between two measurement channels is described below and in the flowchart shown in Figure 3.

1. Divide each channel’s data into segments of duration 0.04s, denoting each segment as x[n]. For example, a 1s recording yields 25 segments per channel.
2. Apply a Hanning window to each segment x[n] to reduce spectral leakage. The Hanning window is chosen for its favorable side-lobe attenuation (−31.5 dB) and moderate main-lobe width (approximately two-FFT-bin increase over non-windowed signal).
3. Compute the DFT of each windowed segment and identify the bin with the highest magnitude (the peak bin).
4. Examine the magnitudes of the bins adjacent to the peak bin to estimate an offset parameter. This offset quantifies how far the true frequency lies from the center of the peak discrete bin using the relative heights of the side bins.
5. Interpolate the DFT using the offset parameter and side-bin magnitudes to estimate the true signal characteristics, including frequency, amplitude, phase, and the DC component.
6. Calculate the ratio between the two channels for each segment using the amplitude and phase values, following the procedure described in Section 3.3.
7. Average the computed ratios across all segments to obtain the overall mean and standard deviation for the measurement.

## 4. Results

### 4.1. Baseline Measurement Setup Results

This section presents the baseline results from the measurement setup, serving as the reference for all the subsequent configuration changes. To ensure reliable comparisons, a new reference measurement was performed daily prior to any setup changes, and the baseline setup remained consistent throughout.

The measurement system uses two simultaneous methods: the primary current (approximately 10 A) and each CT’s secondary current (100 mA) are recorded. The nominal CT ratio is 100:1, but practical effects, such as losses and internal impedance, result in a secondary current that is slightly less than the ideal value. By systematically measuring at discrete frequencies between 50 Hz and 150 kHz, ratio errors can be accurately quantified. The focus is on developing the reference measurement setup, but there is always some interdependence between the CTs used and the measurement. The findings should generalize to other systems, but the exact deviations and uncertainties are determined by the exact components used.

A direct comparison of the secondary currents enables the evaluation of relative CT performance (see Equation (3)). If the CTs have identical frequency responses and there is no capacitive current leakage before the primary current passes through both CTs, the secondary currents will be equal. If any parameter is changed—such as substituting a shunt resistor in one CT—the resulting differences are directly attributable to that change. Additionally, this secondary-to-secondary method is how a DUT CT at higher currents would be characterized. Note that, in this experiment, a DUT or reference CT is not explicitly present. Rather, two CTs of the same model and manufacturer are used. This helps with the development of the measurement method, and in the future can lead to a setup with a reference CT and an unknown DUT.

#### 4.1.1. Current Ratio Comparison

**Figure 4:**
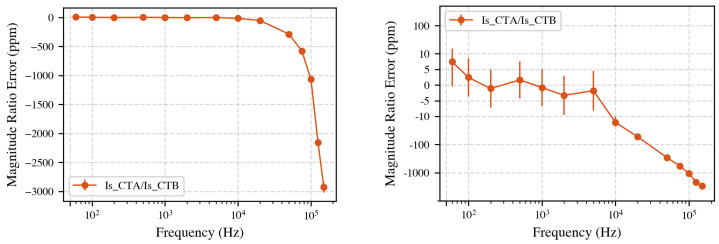
**Left**: Comparison between secondary currents of CT A and CT B. Significant deviations appear above 5 kHz, increasing to 3000 ppm at 150 kHz. **Right**: Symlog-scale shows differences smaller than 5 ppm below 5 kHz; this is within the stated measurement uncertainty.

Figure 4 shows the direct secondary-to-secondary comparison using regular (left) and symmetric logarithmic scale (right). Below 5 kHz, the difference is less than 7 ppm, which is within the stated measurement uncertainty. Above 5 kHz, deviations increase, reaching up to 3000 ppm at 150 kHz. Note that the symlog scale combines linear scaling (between −10 ppm and 10 ppm) with logarithmic scaling elsewhere, highlighting small variations around zero. Note that visually the vertical jump from 5 kHz to 10 kHz may appear similar to the jump from 10 kHz to 50 kHz; however, numerically, the first is about 10 ppm, whereas the second is nearly 500 ppm. Always refer to the regularly scaled plot for accurate interpretation over large frequency ranges.

**Figure 5:**
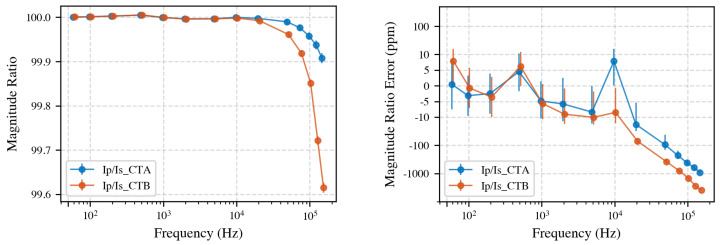
**Left**: Primary-to-secondary current ratios for CT A and CT B. Deviations become more pronounced above 10 kHz. **Right**: Same data plotted on a symmetric logarithmic scale.

Primary-to-secondary current ratio analysis further characterizes the frequency-dependent behavior of the CTs. We compared the measured primary current $I_{p}$ with the secondary currents $I_{s,CTA}$ and $I_{s,CTB}$, the measured ratios across the full frequency range are shown in Figure 5. The power analyzer was calibrated at each discrete measurement frequency for both the 100 mA and 10 A current ranges. Calibration was performed prior to the start of testing using a reference shunt and voltage calibrator system at 50 Hz, achieving an uncertainty of 2.5 ppm [34]. At 150 kHz, the calibration uncertainty increased to approximately 50 ppm. The uncertainty bars shown in the primary-to-secondary ratio plots account for these calibration uncertainties. In the case of secondary-to-secondary measurements, this calibration uncertainty is not included as it is eliminated by interchanging the measurement modules. Up to 10 kHz, the CTs maintain the nominal 100:1 ratio within the measurement uncertainty. Above 10 kHz, ratio errors increase, reaching a deviation of up to 0.4% for CT B at 150 kHz.

Taken together, these results demonstrate that, with the proposed system, CT calibration is feasible up to 150 kHz with less than 0.3% difference between the CTs and less than 0.4% deviation from the ideal ratio. Below 10 kHz, both primary-to-secondary and secondary-to-secondary ratios remain within measurement uncertainty, and, up to 5 kHz, the CTs perform identically.

#### 4.1.2. Phase Comparison of CTs

Accurate phase measurements are essential for power and power quality analysis as even small phase deviations between voltage and current can significantly influence power calculations. Additionally, phase couples into ratio as it is an indication of a filter, which decreases amplitude, with increasing frequency. An important note is that the sampling ammeter was calibrated for absolute amplitude but not for phase; the interchanging technique used in secondary-to-secondary comparisons cancels out systematic phase error between measurement channels. However, an uncalibrated phase offset may remain when comparing primary and secondary currents.

**Figure 6:**
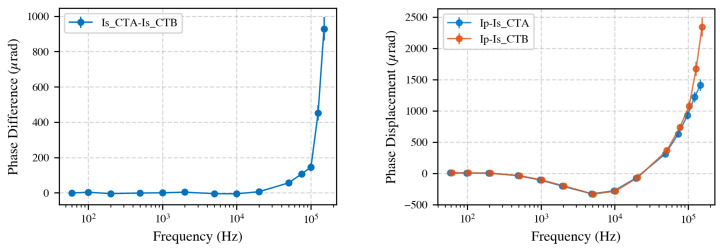
**Left**: Phase differences between secondary currents of CT A and CT B, showing increasing deviations at higher frequencies. Up to 10 kHz, the phase difference between the CTs is less than 10 μrad. **Right**: Phase displacement between primary and secondary currents, where smaller phase shifts for CT A indicate a flatter frequency response at higher frequencies.

Figure 6 presents the phase differences obtained from both comparison methods. On the left, the phase difference between the secondary currents of CT A and CT B is shown. Below 10 kHz, the phase differences are less than 10 μrad, consistent with the findings from the amplitude ratio results. However, at higher frequencies, the phase deviation increases, highlighting differences in the internal electronic design of the CTs.

The right panel of Figure 6 displays the phase displacement between the primary and secondary currents for each CT. In this measurement, CT A shows less phase deviation with increasing frequency than CT B.

In summary, the phase measurements obtained from both methods reinforce the amplitude ratio findings: the CTs are similar at lower frequencies, but differences in both amplitude and phase become increasingly pronounced at higher frequencies. This underscores the importance of broadband characterization of a reference CT for accurate CT calibration.

In the next sections, first some measurement effects of these baseline results are investigated. After that, these results are compared to alternate setup scenarios. The change will always be introduced to CT A, and CT B will be left unchanged. From this, some conclusions can be drawn about what variables influence the ratio of the CTs and thus the calibration of CTs.

### 4.2. Ammeter Warm-Up

To ensure stable and repeatable secondary current ratio measurements, the ammeter and its internal shunts were allowed to warm up for at least 30 min before testing. Both secondary currents were measured using identical shunt types inside the sampling ammeter. It was observed that, when measuring continuously at 150 kHz, the difference between the two shunts could drift by up to 10 ppm over the first hour after power-up. However, after allowing a 30-min warm-up period with the instrument powered on, any further drift was limited to less than 1 ppm at 150 kHz and 0.5 ppm at 50 Hz. For all the measurements in this study, a minimum 30-min warm-up was therefore adopted, minimizing time-dependent errors due to thermal effects in the ammeter shunts.

### 4.3. Averaging Time and Standard Deviation

Once the ammeter and its internal shunt resistor are thermally stable, the measurement standard deviation can be reduced by increasing the averaging time. A 10-s measurement is generally sufficient to achieve ppm-level deviation. This is probably not only because of ‘white noise’ on the measurement but also because of drift and other frequency components coming from the current generation circuit. Additionally, this is dependent on the amplitude determination algorithm used. Alternative algorithms may have superior noise suppression and thus require a shorter measurement for the same standard deviation.

The Allan deviation was evaluated over a 60-s measurement period to quantify this behavior. The observed $\tau^{-1/2}$ confirms that the noise is white and that extended averaging effectively reduces the standard deviation, with no evidence of drift or flicker noise throughout the measurement interval.

### 4.4. Repeatability of the Complete Test Cycle

**Figure 7:**
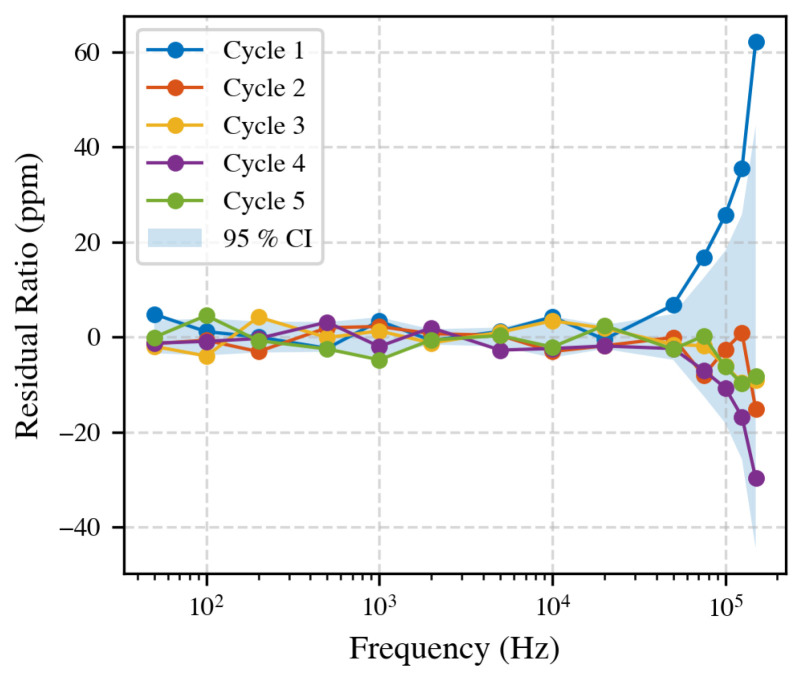
Residual CT ratio over five independent runs. Repeatability is better than ±5 ppm below 20 kHz and increases at higher frequencies.

To ensure the stability of the measurement system, a reference measurement was performed daily. This routine enabled consistent comparison between different test configurations, eliminating long-term drift from the results. However, it is also important to quantify the amount of variation that might occur within a single day. To assess this, the full measurement procedure was repeated five times, each time disconnecting and reconnecting all cables, powering the equipment off and on, allowing a 30-min warm-up, and recording 10 s of data at 14 fixed frequencies using a 10 A current. A 20-min waiting period was included between each cycle to replicate reconfiguring a new setup. Figure 7 shows the residual ratio (deviation from the mean) over five repetitions. Up to 20 kHz, deviations remain within a few ppm; above this, the spread increases.

### 4.5. Grounding and Earth-Loop Effects

**Figure 8:**
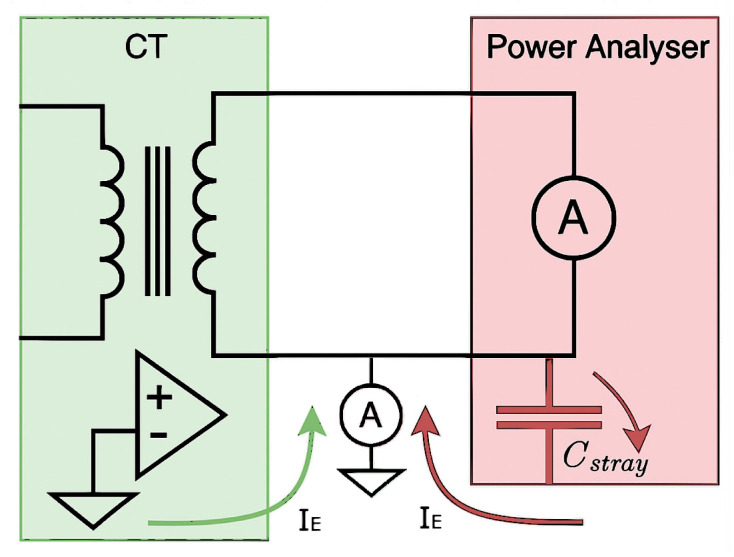
Equivalent schematic illustrating earth-loop current paths in the measurement system. Although ideally isolated, stray capacitances ($C_{\mathrm{stray}}$) and leakage currents through compensation circuits could generate unintended currents via the protective earth (PE), influencing secondary current measurements. One of the modules of the ammeter is inserted between the secondary side of the CT and earth.

Although both the sampling ammeter and the reference current transformers are designed for floating operation, parasitic capacitances between internal circuits and instrument chassis, combined with protective-earth (PE) connections, could introduce unintended earth-loop currents ($I_{E}$). Figure 8 illustrates two possible pathways responsible for these currents:

1. Capacitive coupling between internal circuitry and grounded enclosures.
2. Leakage currents originating from CT compensation electronics referenced to PE.

**Figure 9:**
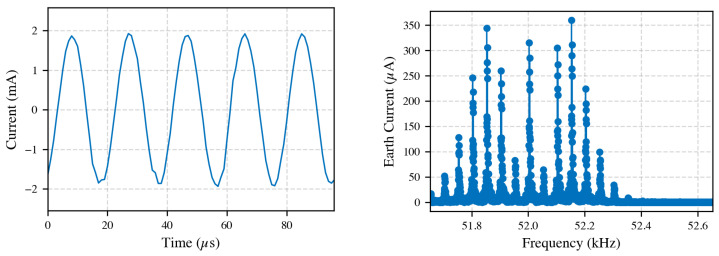
**Left**: Time-domain waveform of earth current. **Right**: Corresponding FFT illustrating broadband frequency content. Measurements performed at 50 Hz primary current, revealing earth-loop currents around 52 kHz.

When applying a primary current at 50 Hz, two distinct groups of earth-loop currents were observed. The first group consists of currents with frequencies around 52 kHz, characterized by a broadband frequency content rather than a single discrete frequency. These frequency components varied intermittently over the 10 s measurement period, with an RMS current of approximately 680 μA in the (52 ± 1) kHz band. The corresponding time-domain and frequency-domain measurements for this earth-loop current at 50 Hz primary excitation are presented in Figure 9.

At higher excitation frequencies, such as 150 kHz, the 52 kHz earth-loop current remained consistent at roughly 680 μA, indicating no significant spectral overlap with the investigated frequencies; the exact source was not identified.

The second group consists of earth-loop currents at the primary excitation frequency. For instance, applying a 50 Hz primary current resulted in an earth-loop current of about 0.4 μA. This type of interference poses potential measurement concerns as it implies current bypassing the measurement shunt in the ammeter. This phenomenon was only reliably detected at frequencies up to 100 Hz. At higher frequencies, however, the noise floor in the frequency spectrum increases, which prevents the detection of earth-loop currents below roughly 10 ppm. Although no direct measurement of earth-loop currents at this level was observed above 100 Hz, a conservative 5 to 10 ppm contribution has been included in the uncertainty budget to account for this measurement limitation.

**Figure 10:**
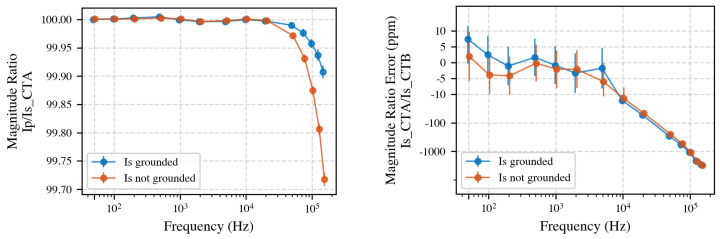
**Left**: Primary-to-secondary current ratio for CT A in grounded versus ungrounded configurations. **Right**: Secondary current ratio between CT A and CT B under both grounding conditions, highlighting minimal discrepancies within measurement uncertainty, except at 50 Hz.

To assess calibration impact, CT ratio measurements were compared between grounded and ungrounded secondary configurations (Figure 10). The observed differences were generally within stated measurement uncertainties, indicating minimal overall influence from grounding. However, at 50 Hz, a statistically significant difference was observed, aligning precisely with the measured earth current of 0.4 μA, corresponding to about 4 ppm in the 100 mA secondary current measurement. This suggests careful consideration for grounding configurations, particularly for high-precision low-frequency measurements.

Removing all earth connections eliminates earth-loop currents but compromises both operational safety and repeatability. Consequently, a single-point grounding scheme was implemented, connecting all the secondary circuits, the ammeter enclosure, and shielding to a common grounding point on the ammeter. This configuration confines earth-loop currents to predictable paths, enhancing safety and measurement consistency.

### 4.6. Selection of Ammeter Shunt

The current measurement ammeter offers three factory-installed shunt resistors, as shown in Table 1.

**Figure 11:**
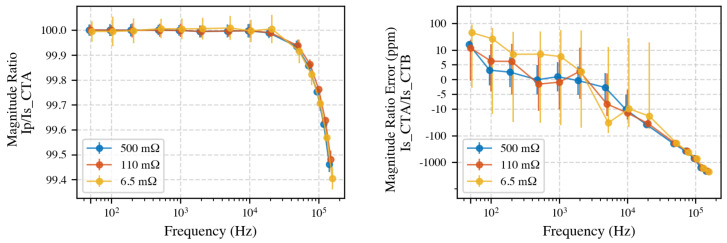
Influence of shunt selection on CT ratio measurements. **Left**: CT A ratio error vs. frequency for all three shunts. **Right**: Inter-CT ratio (B/A) on symlog scale; 6.5 mΩ shunt shows higher uncertainty at low currents. The 110 mΩ shunt provides the best compromise: it loads the CT secondary by only ∼0.5 V at 5 A yet keeps the signal well above the PA’s noise floor during single-tone and two-tone tests.

A lower shunt value minimizes burden on the CT but places the measurement near the power analyzer noise floor; conversely, a higher shunt increases signal magnitude yet loads the CT. Figure 11 (left) compares the Primary-to-secondary ratio of CT A for each shunt and Figure 11 (right) the secondary-to-secondary inter-CT ratio $I_{s\;B}/I_{s\;A}$.

Using the 6.5 mΩ element more than doubles the standard deviation at low secondary currents (<200 mA), whereas the 500 mΩ element is loading the CTs, which is especially an issue for non-compensated CTs, leading to too high a load of 2.5 V. Hence, the 110 mΩ shunt is adopted for all subsequent secondary-current measurements.

### 4.7. Quantization Noise

In a sampling ammeter, there is a limit to the smallest discernible change in the measured quantity of its bipolar analogue-to-digital converter (ADC). For the secondary current measurement, the quantization step size, calculated with the current-domain least-significant bit (LSB), is approximately 1.9 μA (for the 500 mA current range and a front-end 18-bit delta-sigma converter).

Considering that the effective quantization uncertainty is half of an LSB, the effective uncertainty is 0.95 μA. Hence, when measuring the secondary current of 100 mA, this effective quantization uncertainty corresponds to a relative error of approximately 9.5 ppm.

### 4.8. Effect of CT Compensation Electronics

**Figure 12:**
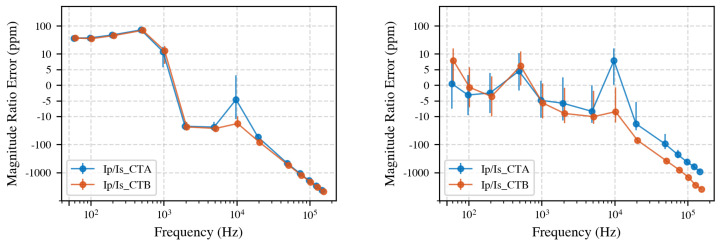
Primary-to-secondary ratio error of CT A and CT B, compensation off (**left**) vs. on (**right**). With compensation disabled, both CTs exhibit an error of 50 ppm to 90 ppm below 1 kHz. Enabling compensation restores the low-frequency ratio to within measurement uncertainty, but diverging amplifier bandwidths cause the two CTs to deviate beyond 20 kHz.

Both NRC CTs employ electronically compensated feedback that injects a correcting current to cancel core magnetization. At higher frequencies, the amplifier bandwidth limits this compensation, potentially affecting the CT’s accuracy. The effect was examined by repeating the primary-to-secondary measurement with the compensation circuitry disabled via the rear-panel, as shown in Figure 12.

Disabling compensation increased the low-frequency ratio error by roughly 50 ppm for both CTs, confirming the efficacy of the feedback network at 50 Hz. Above 20 kHz, however, CT A benefits from a wider-band amplifier, whereas CT B shows negligible improvement. When the electronics are disabled, the CTs behave almost identically. Future designs could explore specially designed high-bandwidth compensation to further flatten the CT response into the supraharmonic range.

### 4.9. Primary-Conductor Positioning

NMIs and industrial calibration laboratories spend effort centering the primary conductor within a CT. If the conductor is off-axis, the magnetic field lines inside the core become non-uniform, potentially driving one sector of the core closer to saturation while another remains less magnetized. It is important to emphasize that the current transformers employed in this study were built for high-accuracy measurements and incorporate several design features to mitigate the influence of external interference and internal coupling effects. Specifically, these CTs do not require a grounded secondary circuit for proper operation, utilize active compensation electronics, and feature carefully spaced secondary windings to minimize capacitive coupling. Additionally, each transformer is housed in a conductive enclosure to suppress external electromagnetic interference.

Voltage-induced leakage currents influence the calibration results of CTs [29,44,45]. In [45], it is shown that this voltage-induced leakage current in the shielded high-voltage cable equals $I_{\mathrm{leak}}=\omega CV$, resulting in a voltage-dependent complex ratio error of a current transformer given by

(7) $$\varepsilon \left(V\right)=\omega C\frac{V}{I_{p}}\sin\varphi \;.$$

Although in our experiments only low voltages are present, at higher frequencies the possibility of capacitive coupling between the primary conductor and the CT secondary winding remains, which could introduce similar errors [46].

For the experimental evaluation, two configurations were compared: centered and eccentric. From the results, no statistically significant deviation is observed up to 150 kHz. Hence, for currents below 10 A and with the adopted 110 mΩ burden, with no additional voltage present, conductor positioning does not measurably affect the ratio for the CTs used in this experiment.

For the CTs used in this experiment, having precisely spaced secondary windings to minimize coupling, an air-gap aperture, and operated with burdens below 0.5 V, the measurements indicate that achieving ppm-level ratio accuracy does not require sub-millimeter centering fixtures. Routine centering to within a few millimeters suffices, simplifying mechanical design for calibration routines.

### 4.10. Mutual Proximity of Two CTs

When two CTs share the same primary conductor during comparison, mutual inductance and stray capacitive coupling between their cores and compensation circuits could, in principle, affect the measured ratio. The CTs used in this study are equipped with a grounded metal shield specifically designed to suppress external flux linkage. However, to verify the effectiveness of this shielding and to quantify any potential proximity effects, the CTs were tested at spacings of 3 cm and 30 cm.

The results show that, up to 10 kHz, there is no observable impact of CT spacing within the measurement uncertainty. At frequencies above 10 kHz, a decrease in the measured secondary current was observed when the CTs were closely spaced, suggesting that some coupling or leakage may occur at higher frequencies.

In this study, a spacing of 30 cm was used for all the subsequent experiments as a practical precaution. It should be noted, however, that this guideline may not generalize to other CT designs, particularly those without dedicated shielding, which may be more susceptible to proximity effects. Additionally, spacing CTs too far apart may introduce capacitive leakage currents from the primary conductor, causing the CTs to no longer measure identical primary currents. Therefore, careful consideration of both proximity and shielding is recommended when designing calibration setups, and validation measurements should be conducted for each new CT type and arrangement.

### 4.11. Cable Characterization

**Figure 13:**
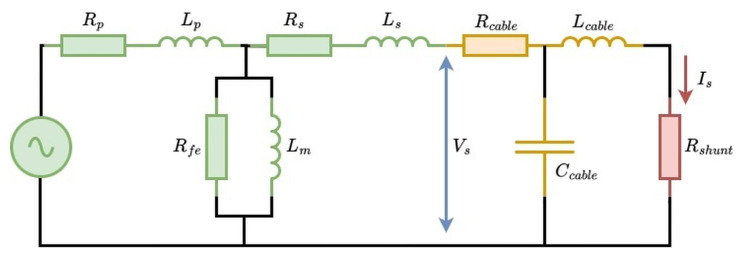
Simplified equivalent circuit depicting the cable measurement setup. The secondary winding of the CT (green) is connected via the test cable (orange) to the internal shunt resistor of the PA (red). Secondary current ($I_{s}$) is determined by measuring the voltage across the PA shunt. Cable characteristics are derived by measuring the voltage ($V_{s}$, blue) at the CT output terminals. In this model, it is assumed that the inductance of the shunt resistor inside the power analyzer is negligible compared to that of the external cable. This assumption occurs as the power analyzer employs internal coaxial shunts specifically engineered to minimize inductance and maintain linearity at higher frequencies.

This section examines the electrical properties of cables linking the current transformer’s secondary side to the current measurement bridge. Secondary side cable selection could influence measurement accuracy, primarily due to the inherent cable impedance. This impedance includes series resistance (*R*), and, for high-frequency measurement, especially important, inductive reactance ($X_{L}$), and potentially parallel parasitic capacitance depending on the cable design. An equivalent circuit illustrating the measurement system is provided in Figure 13.

Cable impedance ($Z_{\mathrm{cable}}$) was determined experimentally by simultaneously capturing voltage ($V_{s}$) and current ($I_{s}$) amplitude and phase. These measurements spanned frequencies from 50 Hz to 150 kHz. Impedance components were computed using phasor notation:

(8) $$Z_{\mathrm{cable}}=\frac{V}{I}\;⟹\;R=ℜ\left(Z_{\mathrm{cable}}\right),\;X_{L}=ℑ\left(Z_{\mathrm{cable}}\right)$$

High-frequency operation of current transformers makes them particularly sensitive to elevated secondary impedance. The CT must produce a sufficient secondary voltage to achieve the specified current ratio. This effect is notably pronounced in electronically compensated CTs since their internal feedback mechanisms inject compensatory current to mitigate leakage. Figure 13 illustrates how secondary impedance prompts the CT to generate voltage across its magnetizing inductance ($L_{m}$) and core losses ($R_{fe}$), thus increasing leakage current through these parallel components.

#### 4.11.1. Twisted-Pair Cable Performance

Twisted-pair stranded cables, preferred for their electromagnetic noise shielding, were assessed for inductance and resistance. The cables used in this study were banana test leads with a 0.75 mm^2^ cross section. At 150 kHz, inductive reactance ($X_{L}$) measurements for lengths 50, 100, and 190 cm showed an inductance of approximately 0.78 μH/m (Table 2). Linear regression confirmed cable inductance linearity with length, yielding $R^{2}=0.99$.

**Figure 14:**
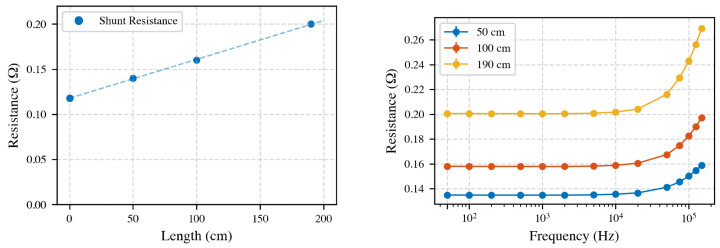
**Left**: Linear correlation between cable length and resistance at 150 kHz, indicating a resistance of 0.04 Ω/m and validating that the other resistance is from the internal shunt of 0.11 Ω. **Right**: Frequency-dependent resistance highlighting increased resistance proportional to cable length. High-frequency effects (skin and proximity) can increase the observed *R* compared to the 50 Hz value).

Resistance measurements corrected for the internal PA shunt (0.110 Ω) indicated cable resistance of about 0.04 Ω/m, confirming negligible additional resistance beyond the known internal shunt, as shown in Figure 14.

#### 4.11.2. Coaxial Cable Performance

**Figure 15:**
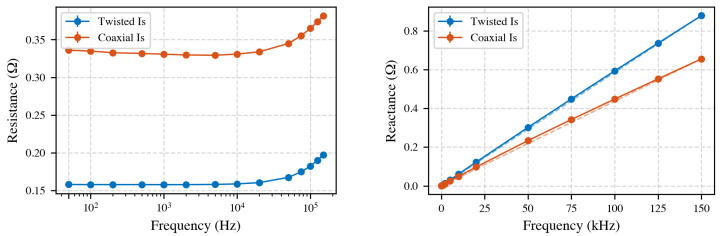
Comparison of coaxial and twisted-pair cables (100 cm) at varying frequencies. **Left**: Higher coaxial cable resistance is evident. **Right**: Inductive reactance ($X_{L}$), indicating linear frequency-dependent inductance.

RG-58 coaxial cables offer shielding and precise impedance control, displaying slightly lower inductance (0.68 μH/m) but significantly higher resistance compared to twisted-pair cables, as shown in Figure 15. Additionally, inherent parallel capacitance in coaxial cables risks accuracy at higher frequencies by bypassing the measurement shunt.

Overall, twisted-pair cables balance lower resistance and manageable inductance, whereas coaxial cables, although lower in inductance, introduce higher resistive and capacitive leakage paths. For primary to secondary current measurements, the choice of cable is dependent on CT secondary voltage capabilities and measurement accuracy requirements. For CT to CT ratios, as long as the cables are reasonably identical, the effects should largely cancel out. However, the signal-to-noise ratio decreases, which can be critical for detecting high-order harmonics with very small amplitudes. Therefore, minimizing current losses helps to improve sensitivity.

### 4.12. Uncertainty Budget

An uncertainty budget was constructed following the GUM guidelines to quantify the uncertainty associated with the CT ratio error measurement system [47]. This budget consolidates the main contributions identified throughout the previous analyses. Table 3 presents the expanded uncertainties (k=2) for the secondary-to-secondary current comparison method. It is important to note that the uncertainty of the reference CT itself is not included; the creation and full characterization of a broadband reference CT are identified as aspects for future work.

In the secondary-to-secondary method, many sources of uncertainty are eliminated because they are common to both CTs. For example, any gain error in the ammeter cancels due to the channel interchange technique. This makes the method robust and particularly suitable for high-accuracy relative measurements. The current setup can also be adapted for primary-to-secondary calibration, where the measurement of primary current introduces additional uncertainty (approximately 2.5 ppm at 50 Hz and increasing to 50 ppm at 150 kHz).

Table 3 highlights the combined expanded uncertainties in the magnitude ratio measurement for three representative frequencies: 50 Hz, 10 kHz, and 150 kHz. Additional frequency points follow similar trends, and their uncertainties are depicted in the relevant result figures.

This uncertainty analysis confirms that, for the secondary-to-secondary comparison method, the combined expanded uncertainty remains below 10 ppm up to 10 kHz, and below 100 ppm at 150 kHz.

## 5. Discussion and Conclusions

This paper presents a simplified calibration method for current transformers (CTs), utilizing a sampling ammeter (power analyzer) to directly measure the ratio of secondary currents, replacing the more complex systems previously employed involving standard transformers, buffering circuits, and voltage samplers. The measurement method simplifies the calibration process, reduces complexity, and directly enables up to 150 kHz bandwidth, covering the IEC WB3 class [12].

The developed calibration setup achieved an expanded measurement uncertainty (k=2) of approximately 10 ppm up to 10 kHz, an improvement with respect to the previous 50 ppm achieved with the same system [27]. Beyond 10 kHz and up to 150 kHz, the magnitude ratio uncertainties remained below 120 ppm, thus outperforming the present state-of-the-art approaches [22,23] and confirming the suitability of this simplified setup for high-frequency characterization. Note that the reference CT itself will introduce an additional magnitude ratio uncertainty of approximately 2.5 ppm at 50 Hz up to 50 ppm at 150 kHz.

Several operational parameters and configuration choices affecting the measurement accuracy and repeatability were also investigated. A typical warm-up period of approximately 30 min for the sampling ammeter was established, effectively limiting temperature-induced drift to approximately 1 ppm. With a measurement sampling duration of 10 s per frequency setpoint, the uncertainty due to sampling was maintained below 5 ppm to 150 kHz. To achieve these uncertainties, daily reference measurements for comparison were performed to account for potential temporal or laboratory-condition-related drifts.

Grounding the secondary circuits of the CTs resulted in a measurable difference of about 4 ppm in the measurement at frequencies up to 100 Hz, whereas no significant impact was observed beyond this frequency. To improve repeatability, grounding was implemented for all the subsequent tests.

The choice of the measurement shunt resistor value plays a significant role in balancing measurement accuracy and averaging time. In this work, a resistance of approximately 110 mΩ was effective, although the optimal value may vary depending on the application and instrument characteristics. The selected value is relatively high for CT calibrations as its burden leads the CT to deliver voltage, which increases magnetization losses. However, given that 1 m of twisted pair cable connected to the secondary side adds an impedance of around 1 Ω at 150 kHz, it is probably an acceptable compromise. Although the electronically compensated CTs in this study did not exhibit measurable sensitivity to cable type, different CT designs may be more susceptible.

The shielded housings of the CTs, when properly grounded, were effective in mitigating interference at close proximity up to 10 kHz; above this, some coupling can be observed. Whereas a spacing of 30 cm between transformers was used for these experiments, validation for different CTs and configurations remains important. The position of the primary conductor did not significantly influence the measurement results for the CTs used in these experiments either.

To demonstrate the applicability of the new setup, in future work, the CTs used in this study will be used as a reference to calibrate other types of CTs, such as those developed in [48]. The influence of a high-current fundamental tone of several hundreds of amperes at 50 Hz can be investigated using a separate primary winding [49]. It may be beneficial to design higher-bandwidth compensation electronics for the reference CTs to flatten their frequency response. This way, the bandwidth of the entire system could be extended to 500 kHz to cover the IEC WB4 class [12] as well.

Further improvements may be realized by optimizing grounding. Measuring earth currents across a range of secondary current levels to determine scaling behavior or isolating the contribution of grounded internal amplifiers within the CTs by disabling or removing them could help to clarify the origin of observed earth loop currents. In addition, the effects of cable geometry and material could be examined, for instance, by using short Litz wires or alternative cable configurations to better understand and manage high-frequency impedance and leakage effects. Capacitive coupling between the primary and secondary windings, particularly at higher voltages at these elevated frequencies, remains a topic for further exploration as it may become more significant under different operating conditions. The impact of electromagnetic interference, which could be the origin of day-to-day fluctuations (the largest uncertainty contribution at the highest frequencies), might be further investigated by changing the input filter settings of the ammeters.

## Figures and Tables

**Table 1:** Current measurement modules.

Module	$R_{\mathrm{sh}}$ (mΩ)	Nominal Range	Coverage in This Work
30 A module	6.5	0–30 A	Primary current (10 A)
5 A module	110	0–5 A	Secondary current (up to 200 mA)
5 A module	500	0–0.5 A	Not used

**Table 2:** Twisted-pair cable reactance and resistance at 150 kHz.

Cable Length (cm)	Reactance *X*_L_ (Ω)	Measured $R_{\mathrm{total}}$ (Ω)	$R_{\mathrm{cable}}$ (Ω)
50	0.52	0.14	0.03
100	0.88	0.16	0.05
190	1.56	0.20	0.09

**Table 3:** Magnitude ratio uncertainty budget for the secondary-to-secondary current CT ratio measurement system. All entries are expanded uncertainties (k=2) in ppm.

Source of Uncertainty	50 Hz	10 kHz	150 kHz
Same-type shunt temperature drift (Section 4.2)	0.5	0.5	1.0
Measurement noise (Section 4.3)	3.0	3.3	10.8
Intra-day repeatability (Section 4.4)	3.1	3.4	96
Grounding/interference (Section 4.5)	4.0	5.0	10.0
Instrument resolution (Section 4.7) ^1^	3.8	3.8	3.8
Combined expanded uncertainty U(k=2)	7.0	7.9	97

^1^ Instrument quantization is modeled as a rectangular distribution ($k=1/\sqrt{3}$). As the errors are correlated, the effective error is further divided by $1/\sqrt{2}$.

## Data Availability

Dataset available on request from the authors.
